# Sequence capture phylogenomics of eyeless *Cicurina* spiders from Texas caves, with emphasis on US federally-endangered species from Bexar County (Araneae, Hahniidae)

**DOI:** 10.3897/zookeys.769.25814

**Published:** 2018-06-26

**Authors:** Marshal Hedin, Shahan Derkarabetian, Jennifer Blair, Pierre Paquin

**Affiliations:** 1 Department of Biology, San Diego State University, 5500 Campanile Drive, San Diego CA, 92182, USA; 2 Department of Organismic and Evolutionary Biology, Museum of Comparative Zoology, Harvard University, 26 Oxford St., Cambridge MA, 02138, USA; 3 Blair Wildlife Consulting, 3815 Dacy Lane, Kyle TX, 78640, USA; 4 Scienceinfuse Inc, 12 Saxby Sud, Shefford, QC, J2M 1S2, Canada

**Keywords:** cave evolution, conservation, karst, mitochondrial by-catch, taxonomy, ultra-conserved element

## Abstract

Morphological, mitochondrial, and nuclear phylogenomic data were combined to address phylogenetic and species delimitation questions in cave-limited *Cicurina* spiders from central Texas. Special effort was focused on specimens and cave locations in the San Antonio region (Bexar County), home to four eyeless species listed as US Federally Endangered. Sequence capture experiments resulted in the recovery of ~200–400 homologous ultra-conserved element (UCE) nuclear loci across taxa, and nearly complete COI mitochondrial DNA sequences from the same set of individuals. Some of these nuclear and mitochondrial sequences were recovered from “standard” museum specimens without special preservation of DNA material, including museum specimens preserved in the 1990s. Multiple phylogenetic analyses of the UCE data agree in the recovery of two major lineages of eyeless *Cicurina* in Texas. These lineages also differ in mitochondrial clade membership, female genitalic morphology, degree of troglomorphy (as measured by relative leg length), and are mostly allopatric across much of Texas. Rare sympatry was confirmed in Bexar County, where members of the two major clades sometimes co-exist in the same karst feature. Both nuclear phylogenomic and mitochondrial data indicate the existence of undescribed species from the San Antonio region, although further sampling and collection of adult specimens is needed to explicitly test these hypotheses. Our data support the two following species synonymies (*Cicurina venii* Gertsch, 1992 = *Cicurina madla* Gertsch, 1992; *Cicurina loftini* Cokendolpher, 2004 = *Cicurina vespera* Gertsch, 1992), formally proposed here. Overall, our taxonomy-focused research has many important conservation implications, and again highlights the fundamental importance of robust taxonomy in conservation research.

## Introduction

The limestone cave and karst habitats of Texas are home to hundreds of endemic cave-obligate animal species, including many eyeless spider species. The spider subgenus Cicurella (genus *Cicurina*) includes 60 described species, almost all endemic to Texas caves (Gertsch 1992, Cokendolpher 2004, Paquin and Dupérré 2009). Four eyeless Texas *Cicurina* species, all from Bexar County in the vicinity of San Antonio, are listed as US Federally Endangered (Service 2000). The listed species include *C. madla*, *C. baronia*, *C. vespera* and *C. venii*, the latter three of which are hypothesized single-cave endemic species. The conservation issues faced by these range-restricted taxa are unquestionable and ongoing, and include such threats as habitat destruction, chemical contamination, and invasive species (Service 2011).

Many authors have discussed the challenges of phylogenetic and taxonomic research in Texas cave *Cicurina* (Cokendolpher 2004, Paquin and Hedin 2004, Paquin et al. 2008, Paquin and Dupérré 2009, Hedin 2015). About 90% of collected specimens are immatures, and almost all adult specimens are females (Cokendolpher 2004, Paquin and Dupérré 2009). Given adult male rarity, males cannot be used reliably in species identification. Instead, adult females are primarily used for taxonomic decisions, but female genitalic morphology is variable both within and among caves, blurring the distinction between geographic variation and species level divergence (Paquin et al. 2008, Paquin and Dupérré 2009, Hedin 2015). This indistinct boundary between geographic variation and species level divergence also extends to the genome, because in naturally-fragmented karst habitats some level of genetic population structuring is an expectation (Hedin 2015). Finally, access to Texas caves is difficult, leading to small sample sizes and geographic sampling gaps, both of which impact phylogenetics and species delimitation, particularly molecular species delimitation (e.g., Niemiller et al. 2012, Carstens et al. 2013, Satler et al. 2013).

The special challenge of species delimitation in Texas cave *Cicurina* is exemplified by *Cicurina venii*. This federally endangered species is known only from a single adult female from the type locality (Bracken Bat Cave), the entrance to which has been buried since about 1990 (Cokendolpher 2004). Here is an example of an extremely important species hypothesis that is founded on fundamentally limited data, where the probability of sampling additional specimens (if the type population still persists, which is itself unknown) is very low because of habitat inaccessibility.

Hedin (2015) suggested that next-generation sequencing (NGS) methods, such as sequence capture of ultra-conserved elements (UCEs; ultraconserved.org/), might be used to help resolve challenging phylogenetic and taxonomic problems in eyeless *Cicurina*. The availability of conserved UCE probes makes it possible to capture hundreds of orthologous nuclear genetic regions (“loci”) from a set of specimens using cost-effective, scalable methods (Faircloth et al. 2012, Bossert and Danforth 2018). Within and between closely-related species, most phylogenetic information is coming from variable regions that flank the core UCE, and several recent publications have shown that such flanking regions carry enough phylogenetic information to robustly resolve species-level divergences (e.g., Smith et al. 2014, Blaimer et al. 2016a,b, McCormack et al. 2016, Zarza et al. 2016, Newman and Austin 2016, Starrett et al. 2017). Another potential benefit of UCE capture is that major portions of the mitochondrial genome are often included as “by-catch” in sequence reads (e.g., Zarza et al. 2016), further increasing phylogenetic return on investment.

An additional appeal of UCE-based phylogenomics is that the method has demonstrated applicability with degraded DNA (e.g., on “standard” museum specimens, without special preservation of DNA material). For example, hundreds or thousands of UCE loci have been captured from old bird museum specimens (McCormack et al. 2016), formalin-fixed snakes (Ruane and Austin 2017), and pinned insects (Blaimer et al. 2016a). Recently, Hedin et al. (2018) extended this utility to “standard” ethanol-preserved spider museum specimens. This type of utility would apply importantly to Texas cave *Cicurina*, as multiple regional collections house large numbers of both adult and immature specimens, many collected in the past 20–30 years. Given the natural rarity of eyeless *Cicurina* spiders, and general difficulty of cave access, the ability to use historical collections for sub-genomic phylogenomics could prove transformative. Here we combine morphological, mitochondrial, and UCE nuclear data to address phylogenetic and species delimitation questions in Texas cave *Cicurina*, focusing specifically on federally listed taxa from Bexar County.

## Materials and methods

### UCE specimen sampling and DNA extraction

Specimens and/or DNA extractions were made available from multiple institutions and persons, including the Texas Memorial Museum (TMM), Texas Tech University (TTU), ZARA Environmental, and the American Museum of Natural History (AMNH) (see Acknowledgements). In total, we attempted to gather UCE data for 83 eyeless *Cicurina* specimens, six of which were “standard” museum specimens (without special preservation of DNA material, Suppl. material 5), including holotype specimens of *C. venii* and *C. vespera*. Our sample emphasized species and populations from Bexar County, but we also included samples opportunistically from caves in counties to the northeast and west of San Antonio.

Many specimens used in this study were immatures, but could be tentatively identified to species in a *post hoc* manner based on phylogenetic placement into genetic clades including adult specimens, bolstered by locality data (i.e., caves from which adult *Cicurina* specimens have been collected in the past, including type locations). The assumption of no sympatry is fundamentally important here (i.e., one eyeless *Cicurina* species per cave). No sympatry is the rule for these spiders (Cokendolpher 2004, Paquin and Dupérré 2009), but as we show here (see Results), rare sympatry does occur. We acknowledge that the use of immatures is suboptimal, but in this system represents a clear trade-off. The inclusion of immatures brings some level of uncertainty, but exclusion results in a dramatic loss of information, again because immatures represent the bulk of collected specimens (Cokendolpher 2004, Paquin and Dupérré 2009).

For specimens preserved for DNA studies (preserved in high percentage ethyl alcohol at -80 °C), genomic DNA was extracted from leg tissue using the Qiagen DNeasy Blood and Tissue Kit (Qiagen, Valencia, CA). For museum samples preserved in 70–80% EtOH we used standard phenol/chloroform extractions with a 24-hour incubation. All extractions were quantified using a Qubit Fluorometer (Life Technologies, Inc.) and quality was assessed via gel electrophoresis on a 1% agarose gel.

### UCE data collection and matrix assembly

UCE data were collected in multiple library preparation and sequencing experiments. Up to 500 ng of genomic DNA was sonicated using a Covaris M220 Focused-ultrasonicator with treatment time of 60–65 s, Peak Incident Power of 50, 10% Duty Factor, and 200 cycles per burst. All museum samples were sonicated for 30 seconds using the same settings. Samples were electrophoresed on agarose gels to verify sonication success.

Library preparation followed Starrett et al. (2017), with minor modifications. Briefly, libraries were prepared using the KAPA Hyper Prep Kit (Kapa Biosystems), using up to 250 ng DNA (i.e., half reaction of manufacturer’s protocol) as starting material. Ampure XP beads (Beckman Coulter) were used for all cleanup steps. For samples containing <250 ng total DNA, all available DNA was used in library preparation. After end-repair and A-tailing, universal adapters were ligated onto libraries at varying concentrations depending on amount of input DNA. Libraries were then amplified in a 50 μl reaction, with 15 μl adapter-ligated DNA, 1X HiFi HotStart ReadyMix, and 0.5 μM of each Illumina TruSeq dual-indexed primer (i5 and i7) with modiﬁed 8-bp indexes (Glenn et al. 2016). Amplification conditions were 98 °C for 45 s, then 18 cycles of 98 °C for 15 s, 60 °C for 30 s, and 72 °C for 60 s, followed by a final extension of 72 °C for 60 s. Samples were quantified again to ensure amplification success, and equimolar amounts of libraries were combined into 1000 ng total pools consisting of eight samples each (125 ng per sample).

Target enrichment was performed on pooled libraries using the MYbaits Arachnida 1.1K version 1 kit (Arbor Biosciences; Faircloth 2017) following the Target Enrichment of Illumina Libraries v. 1.5 protocol (http://ultraconserved.org/#protocols). Hybridization was conducted at 60 or 65 °C for 24 hours. Following hybridization, pools were amplified in a 50 μl reaction consisting of 15 μl of hybridized pools, 1X Kapa HiFi HotStart ReadyMix, and 0.25 μM of each of TruSeq forward and reverse primers. Amplification conditions consisted of 98 °C for 45 s, then 16 or 18 cycles of 98 °C for 15 s, 60 °C for 30 s, and 72 °C for 60 s, followed by a final extension of 72 °C for 5 minutes. Following an additional cleanup, libraries were quantified using a Qubit fluorometer and equimolar mixes were prepared for sequencing on an Illumina HiSeq 2500 (Brigham Young University DNA Sequencing Center) using 125 bp PE reads.

Raw demultiplexed reads were processed using the Phyluce pipeline (Faircloth 2016). Quality control and adapter removal were conducted with the Illumiprocessor wrapper (Faircloth 2013). Assemblies were created with Velvet (Zerbino and Birney 2008) using default settings. Contigs were matched to probes using minimum coverage and minimum identity values of 65. UCE loci were aligned with MAFFT (Katoh and Standley 2013) and trimmed with Gblocks (Castresana 2000; Talavera and Castresana 2007) implemented in the Phyluce pipeline.

Individual UCE loci were imported into Geneious 10.1 (Biomatters Ltd.) for manual inspection. In particular, alignments with low % identical sites (less than 40%) were flagged for inspection. If exclusion of a single divergent sequence increased this value to > 75%, the locus was retained. Subsequently, all loci were inspected - individual sequences with large gaps in the core UCE region were excluded, and obvious alignment errors in flanking regions were manually adjusted.

### UCE phylogenomic analyses

Concatenated data matrices with 50% and 70% occupancy (i.e., for any given locus, sequences for at least 50 or 70% of samples needed for locus inclusion in the final dataset) were assembled for phylogenomic analyses. Maximum likelihood analyses of both matrices were conducted using RAxML version 8.2 (Stamatakis 2014) with the GTRGAMMA model and 200 rapid bootstrap replicates. Bayesian analyses were run on the concatenated 70% matrix using BEAST 2.4.0 (Bouckaert et al. 2014) with the molecular model estimated using PartitionFinder 1.1.1 (Lanfear et al. 2012). This analysis was run for 10 million generations, logging every 1000. Stationarity was assessed using Tracer (Rambaut and Drummond 2007) to ensure all ESS values were above 200. Two coalescent analyses were also conducted for both 50% and 70% matrices. First, ASTRAL-II (Mirarab et al. 2014; Mirarab and Warnow 2015) was used with individual gene trees estimated in RAxML with 500 bootstrap replicates. We also used SVDquartets (Chifman and Kubatko 2014, 2015) with n = 500 bootstraps, as implemented in PAUP* 4.0 (Swofford 2003). RAxML phylogenies were midpoint rooted, while BEAST trees were rooted according to the implemented clock model.

### Mitochondrial phylogenetics

Using a sample of eyed and eyeless *Cicurina* spiders from Texas and other US states, Paquin and Hedin (2004) recovered a “Texas eyeless” mitochondrial cytochrome oxidase I (COI) clade. We downloaded representative sequences for this clade from GenBank. We also used Geneious BLAST searches to extract COI sequences from UCE Velvet assemblies, using Texas eyeless clade Sanger data as query sequences (blastn, no low complexity filter, max e-value of 1e-5). For any single specimen these searches sometimes returned multiple sequences covering the same region; here, large differences in contig coverage values were used to discard putatively non-homologous contigs. After assembly of the Sanger plus UCE “by-catch” matrix (984 basepairs × 132 taxa), a partitioned (by codon position) RAxML analysis was conducted with the GTRGAMMA model and 200 rapid bootstrap replicates. Following Paquin and Hedin (2004), we rooted the mitochondrial tree using sequences from *Cicurina pampa*, a six-eyed species from Texas.

### Morphological study

Cokendolpher (2004) showed that cave-dwelling *Cicurina* from Bexar County fall into two distinct morphological groups that differ in degree of troglomorphy, as measured by the ratio of first leg length / carapace length. Species with a high “troglomorphy index” (TI) are relatively long-legged as compared to taxa with a lower TI. Although Cokendolpher (2004) measured only adult specimens, we wondered whether this TI difference also applied to immatures. Focusing on Bexar County taxa, we measured adult and immature specimens from multiple species (see Suppl. material 6). Measurements were taken using an Olympus SZ40 dissecting scope fitted with an ocular micrometer, as specified in Suppl. material 6.

For adult specimens used in UCE experiments we imaged genitalia using a Visionary Digital BK plus system (http://www.visionarydigital.com). Individual images were merged into a composite image using Helicon Focus 6.2.2 software (http://www.heliconsoft.com/heliconfocus.html). Because we did not have access to all specimens borrowed from TTU (some loans were DNA only), we were not able to image all adult specimens used in this study.

## Results

Suppl. material 5 includes specimen voucher information, DNA quantities, number of raw Illumina reads passing quality filter, Velvet contig numbers, and SRA accession numbers. We generated UCE data for six total museum specimens, but BLAST searches of both raw reads and Velvet contigs for the *C. venii* and *C. vespera* holotype specimens returned only bacterial, fungal, or human sequences. For 81 remaining specimens we analyzed both 50% (399 loci, 94379 basepairs) and 70% occupancy (238 loci, 61054 basepairs) nuclear UCE matrices (Suppl. material 5). Raw Illumina reads are available at the NCBI Short-Read Archive (BioProject PRJNA471846), with aligned matrices and .tre files available at Dryad (https://doi.org/10.5061/dryad.28fg251).

**Figure 1.**
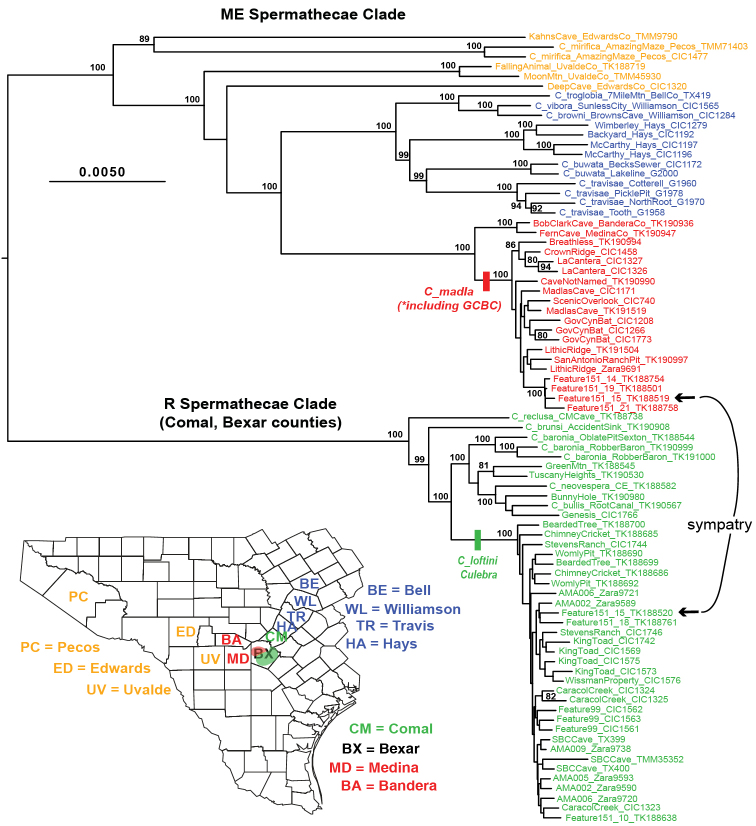
Phylogenetic tree from RAxML analysis of 399 UCE loci, 70% (399_70) occupancy matrix. Bootstrap values below 75 not shown. Geography and morphology highlighted. Abbreviations: ME (= mostly elongate) spermathecae clade, R (= rounded) spermathecae clade. Further details regarding specimen codes and locations found in Suppl. material 5. Inset: sampled Texas counties.

**Figure 2.**
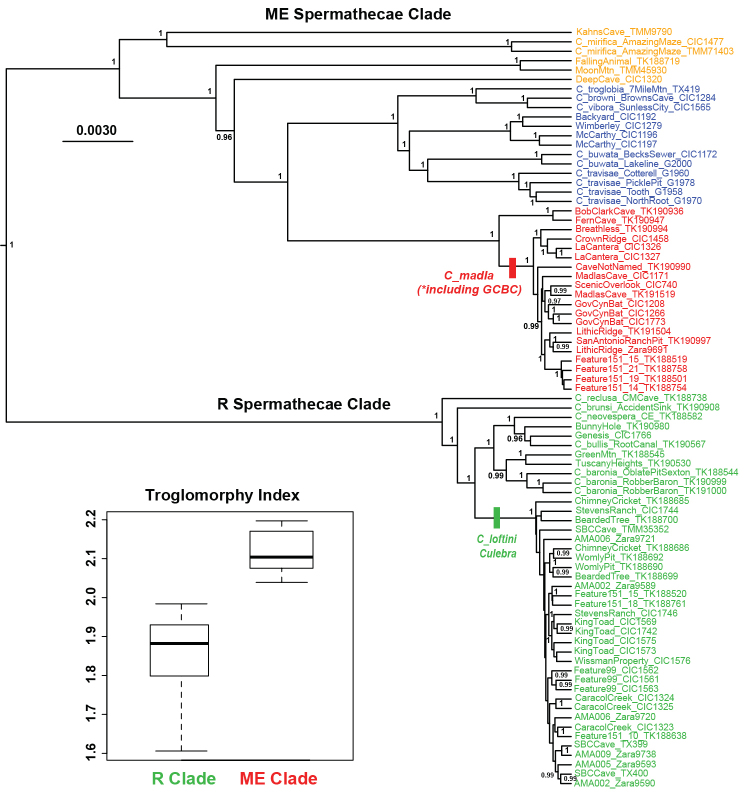
Phylogenetic tree from BEAST analysis of 399_70 UCE matrix. Posterior probability values above 0.95 shown. Inset: Boxplots of TI values (from Suppl. material 6) for ME and R clade members from Bexar County (for ME clade, two small juveniles and Stahl Cave specimens not included).

Using molecular clock or mid-point rooting, all nuclear phylogenomic analyses result in recovery of two primary Texas eyeless *Cicurina* clades (Figures 1, 2; Suppl. material 1–3). A relatively broadly distributed clade includes species with adult females possessing mostly elongate spermathecae (see Cokendolpher 2004, Paquin and Dupérré 2009), hereafter called the ME clade. This clade is found in multiple Texas counties that include Edwards Plateau caves (Figure 1). A more narrowly distributed clade includes species with adult females possessing rounded spermathecae (Cokendolpher 2004, Paquin and Dupérré 2009), hereafter called the R clade. Given our taxon sample, the R clade appears restricted to caves in Bexar and adjacent southern Comal counties (Figure 1).

**Figure 3.**
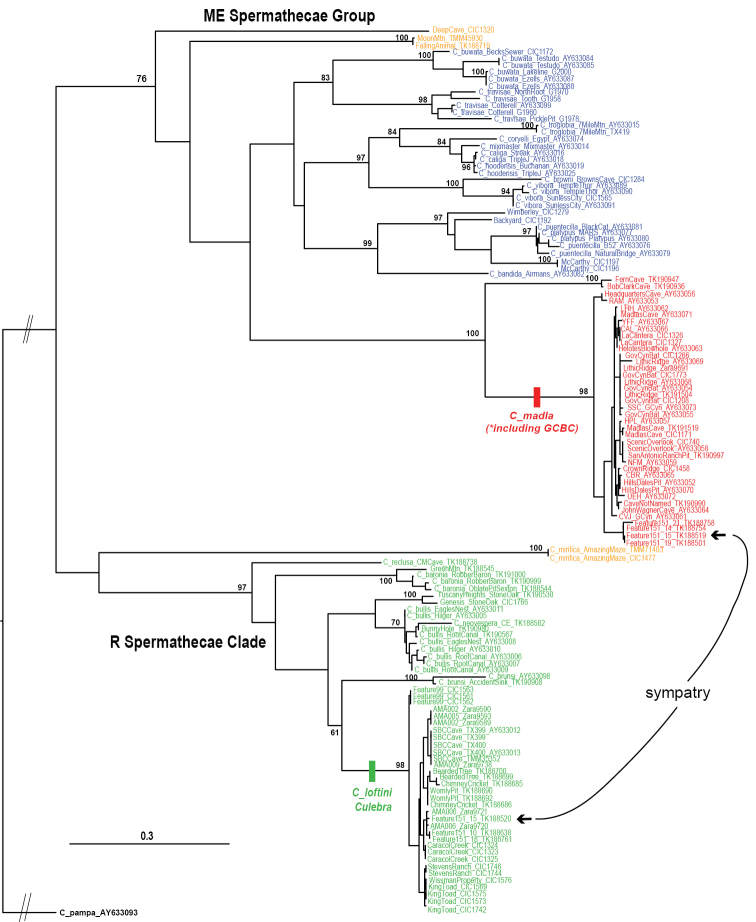
Phylogenetic tree from RAxML analysis of COI mitochondrial data. Previously published sequences with corresponding GenBank numbers (AY#), some with cave location codes as in Paquin and Hedin (2004). Bootstrap values above 70 shown only for major lineages. Bootstrap values within *C. madla* shown on Figure 6, those for *C. loftini* shown on Figure 10.

We recovered mitochondrial COI sequences from UCE assemblies for all but two specimens (TMM_9790, TK_190994), with an overall matrix completeness above 97%. All UCE-derived COI sequences were in frame and lacked stop codons or ambiguities, and when directly compared to previously published Sanger data (e.g., from same cave or sometimes same specimen), were found to be identical (Figure 3). The aligned COI matrix is available at Dryad (https://doi.org/10.5061/dryad.28fg251).

Maximum likelihood trees, rooted with the eyed taxon *C. pampa*, include an R clade, but the poorly supported placement of two ME sequences renders this latter group paraphyletic (Figure 3). We note that both ME and R clades were recovered in Paquin and Hedin (2004), although with more limited taxon sampling. The COI dataset includes some species and populations not included in nuclear analyses, including *C. puentecilla* and *C. platypus*, placed into the mitochondrial ME group. Cokendolpher (2004) discussed the unique morphology of *C. platypus* spermathecae, perhaps best described as a third morphological type (large, even-sized, rounded); *C. puentecilla* has a genital morphology very similar to *C. platypus* (Paquin and Dupérré 2009). The COI phylogeny includes higher resolution (not necessarily accuracy) within some species, but generally lacks support at deeper nodes (Figure 3). As such, most discussion below focuses on the highly supported nuclear UCE results.

**Figure 4.**
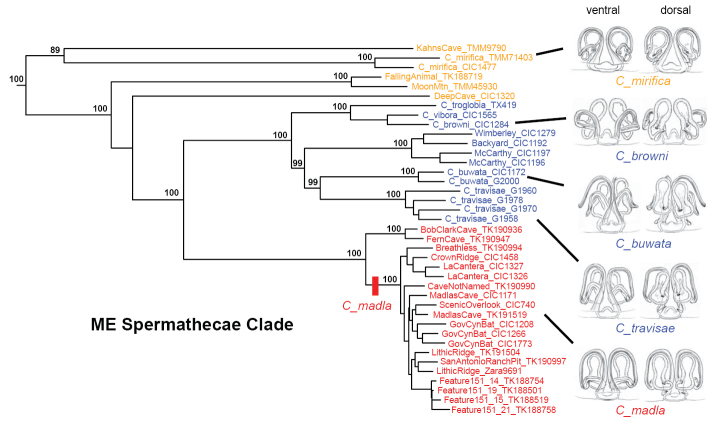
Morphology of ME clade with corresponding 399_70 UCE RAxML phylogeny (not all bootstrap values shown). Holotype female spermathecal images from Paquin and Dupérré (2009), used with permission. Images not to scale.

**Figure 5.**
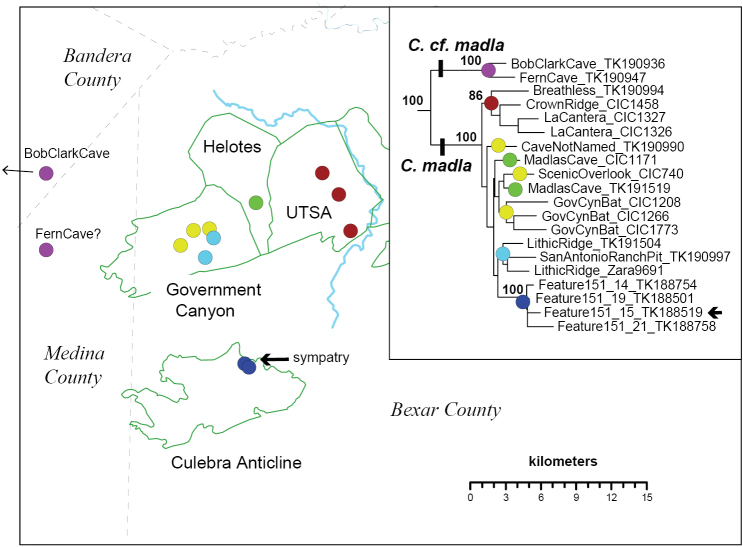
Distribution of *C. madla* and *C. cf. madla* in Bexar and adjacent counties, with corresponding 399_70 RAxML UCE phylogeny (not all bootstrap values shown). Different geographic populations designated by different colors. Cave locations approximate.

Within the ME clade, nuclear phylogenomic relationships are structured geographically, with western, northeastern, and Bexar county groupings (Figure 1). Female morphology is mostly consistent across these groups (Figure 4), and we predict that described *Cicurina* species with similar genital morphologies not sampled here will ultimately fall into the ME genetic clade (e.g., *C. sansaba*, many species from western counties, etc.; see Paquin and Dupérré 2009). Figure 5 shows the distribution of *C. madla* and *C. cf. madla* in Bexar and adjacent Medina and Bandera counties. Consistent with Paquin and Hedin (2004) and new mitochondrial results, sampled populations of *C. madla* are found in multiple karst faunal regions (KFRs) of northwestern Bexar county. A novel result is the placement of multiple samples from the northern Culebra Anticline KFR in the *C. madla* clade (Figure 5). This unexpected distribution across disjunct and geologically isolated KFRs is also seen in the eyeless leptonetid spider *Tayshaneta whitei* (Ledford et al. 2012, fig. 63). At one location in the northern Culebra Anticline (location = “feature 151_15”), members of ME and R clades are found in apparent sympatry, rare in TX cave *Cicurina* (Cokendolpher 2004, Paquin and Dupérré 2009).

**Figure 6.**
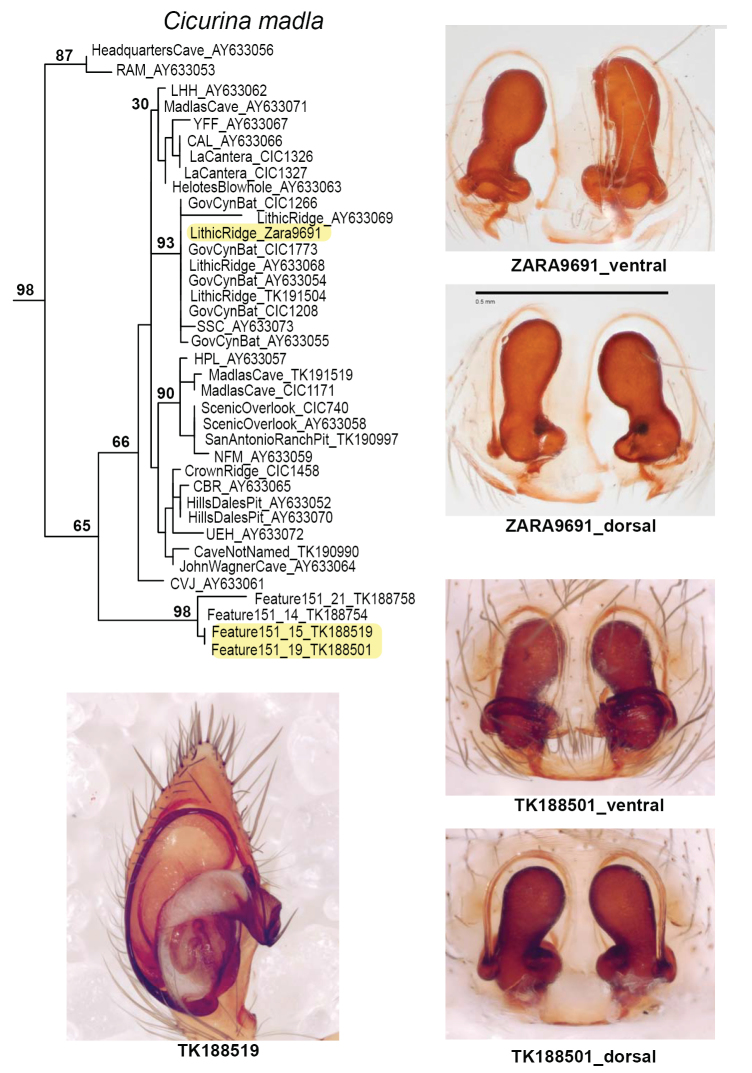
Morphology of adult *C. madla* with corresponding COI RAxML phylogeny. Previously published sequences with corresponding GenBank numbers (AY#), some with cave location codes as in Paquin and Hedin (2004). Imaged specimens highlighted on phylogeny; images of specimens TK_188519 and TK_188501 from Joel Ledford. Scale bar: 0.5 mm.

Figure 6 shows the morphology of adult specimens from the *C. madla* clade, including specimens from the sympatric location. This morphology is consistent with the description for *C. madla*. We note that many additional adult *C. madla* specimens have been collected from sampled (or nearby sampled) caves not shown here (Cokendolpher 2004, Paquin and Dupérré 2009).

We gathered nuclear UCE data for three specimens from Government Canyon Bat Cave (GCBC, the stated type locality of *C. vespera*), all of which fall into the *C. madla* UCE genetic clade. This nesting of GCBC specimens inside a *C. madla* clade also applies to the COI dataset, which also includes two additional Sanger GCBC specimens (Figure 6). In total, five specimens from GCBC have been included in this or prior genetic studies (Paquin and Hedin 2004), and all are genetically allied with *C. madla*. As extensively discussed below, the *C. vespera* holotype specimen (with rounded spermathecae) is morphologically very different from ME spermathecae *C. madla*, and this situation requires special explanation.

**Figure 7.**
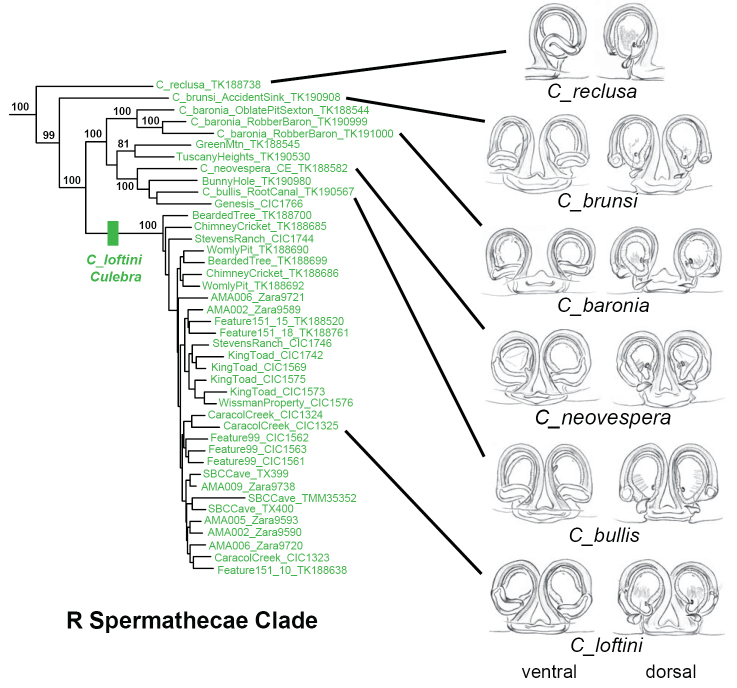
Morphology of R clade with corresponding 399_70 UCE RAxML phylogeny (not all bootstrap values shown). Holotype female spermathecal images from Paquin and Dupérré (2009), used with permission. Images not to scale.

**Figure 8.**
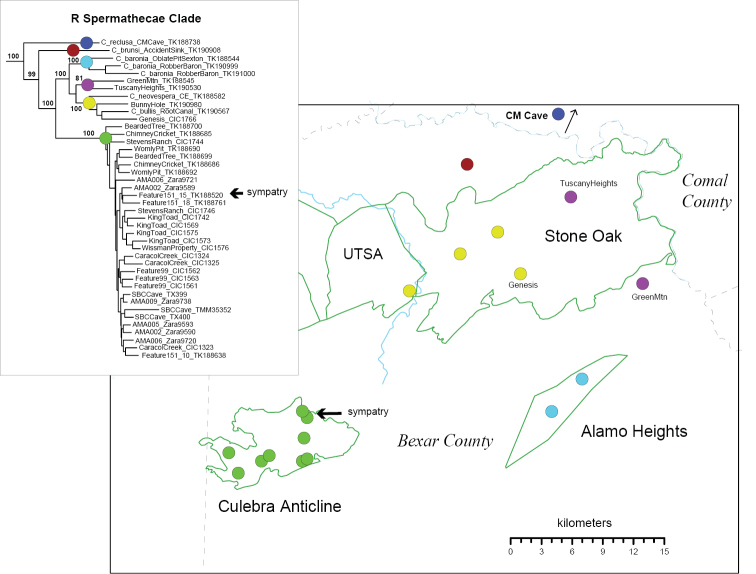
Distribution of R clade in Bexar and Comal counties, with corresponding 399_70 UCE RAxML phylogeny. Different species designated by different colors. Cave locations approximate.

The R clade includes six described species with very similar genitalic morphologies (Figure 7), with a distribution that spans from southern Comal to southwestern Bexar County (Figure 8). These nuclear phylogenomic results agree with Cokendolpher’s (2004) hypothesis regarding the morphological affinities of R clade members, although UCE data do not recover his internal groupings of *C. baronia*, *C. brunsi*, *C. bullis* (rounded spermathecal lobes of unequal size; spermathecal stalk transverse) versus *C. loftini*, *C. neovespera*, and *C. vespera* (rounded spermathecal lobes of unequal size; spermathecal stalk oblique). Both UCE and mitochondrial results confirm a new population for *C. baronia* (=OblatePitSexton), which is important because this Federally endangered species was previously known only from the highly impacted type locality. One area of uncertainty in the R clade involves the species status and phylogenetic placement of specimens from Genesis, GreenMtn and Tuscany Heights locations, with different placements across nuclear and COI analyses (Figure 8; Suppl. material 4).

**Figure 9.**
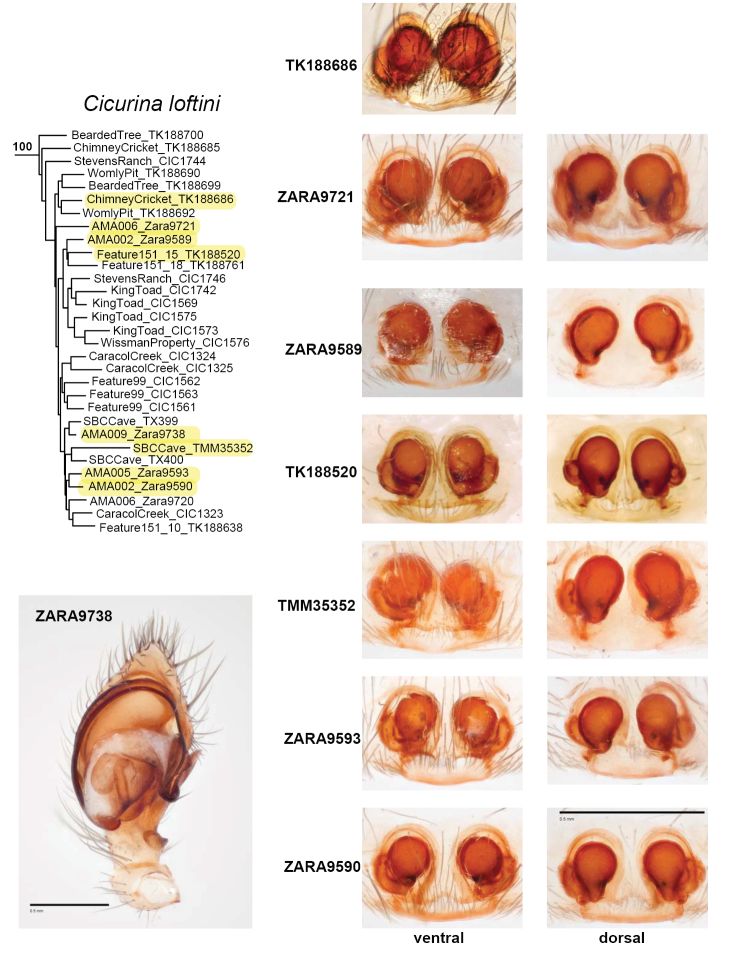
Morphology of adult *C. loftini* with corresponding 399_70 UCE RAxML phylogeny. Imaged specimens highlighted on phylogeny; images of specimen TK_188520 from J. Ledford, specimen TK_188686 from J. Cokendolpher (via Symbiota Collections of Arthropods Network). Scale bar: 0.5 mm.

**Figure 10.**
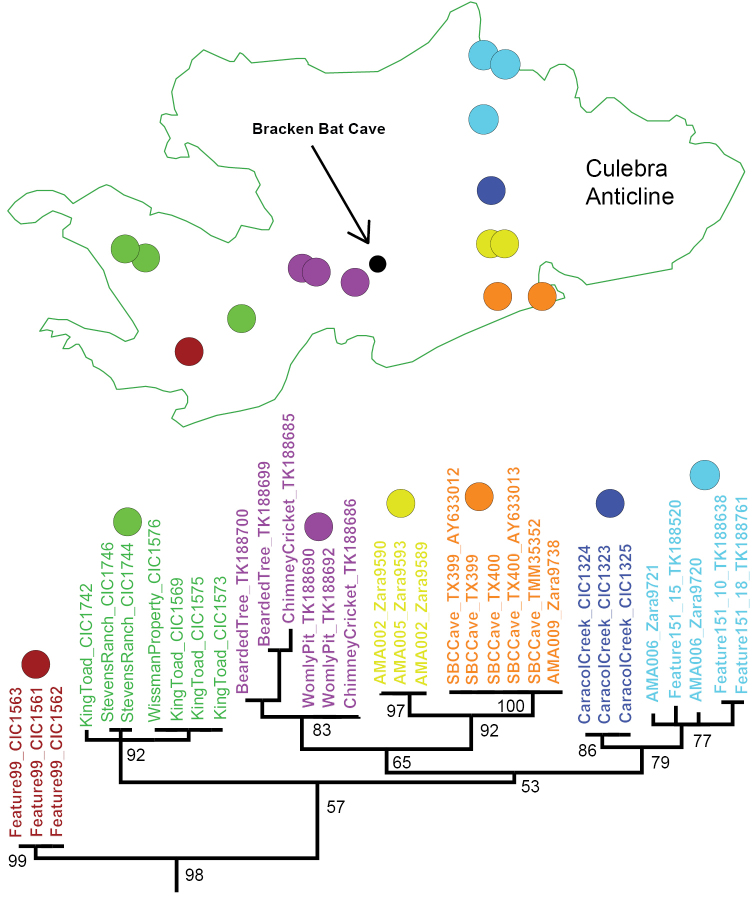
Distribution of *C. loftini* in Culebra Anticline KFR, with corresponding COI RAxML phylogeny. Different mtDNA microclades designated by different colors. Cave locations approximate.

*Cicurina loftini* is found only in the Culebra Anticline KFR (Figure 8). Some populations at the northern edge of this range are in sympatry or close parapatry with *C. madla*, as highlighted in the text above. Figure 9 shows the morphology of adult specimens from *C. loftini*, including adult specimens from the sympatric location; this morphology is consistent with the description for *C. loftini* (Cokendolpher 2004, Paquin and Dupérré 2009). Fine-scale mitochondrial structuring within *C. loftini* appears to closely follow karst geology within the Culebra Anticline KFR, as individual or adjacent caves form distinct and well-supported mitochondrial subclades (Figure 10).

We measured TI values for 49 total specimens, some of which were included in UCE experiments (Suppl. material 6). For 23 specimens representing the R clade, all measured specimens have a TI value (= partial leg I length / carapace length) below 1.98 (Figure 2). This is consistent with the measurements of Cokendolpher (2004, table 1) for *C. baronia*, *C. brunsi*, *C. bullis*, *C. loftini*, *C. neovespera*, and the *C. vespera* holotype, all with TI values below 1.98.

For 25 specimens representing the ME clade, all but two specimens have TI values above 2.04 (Figure 2), consistent with the measurements of Cokendolpher (2004, table 1) for *C. madla* and the *C. venii* holotype. This relatively high TI does not pertain for two very small immature specimens (carapace length below 1 mm) that are suspected members of the ME clade, based on cave location (Suppl. material 6). Also, we measured two specimens from GCBC (different from genetic specimens), the stated type locality of the R morphology, low TI *C. vespera* holotype specimen. Both of these specimens have a TI value above 2.08 (Suppl. material 6). A single immature specimen from Stahl Cave is potentially anomalous. This specimen is from the type locality of the R clade member *C. brunsi*, but has a high TI (2.34) indicative of an ME species (Suppl. material 6). We hypothesize that this represents a potential case of previously unreported sympatry; generation of UCE data for this specimen, or further collecting from this location would provide a test of this hypothesis.

## Discussion

The spider genus *Cicurina* includes over 130 described species known from multiple regions in the northern hemisphere (World Spider Catalog 2018), with many taxa showing troglomorphic modifications associated with cave life (Paquin and Dupérré 2009). It was not our goal to conduct a generic-level phylogenetic analysis, and we acknowledge that we have largely assumed the monophyly of a “Texas eyeless” lineage, as found in the molecular phylogenetic research of Paquin and Hedin (2004), and supported by morphology (subgenus Cicurella Chamberlin and Ivie, Gertsch 1992, Cokendolpher 2004, Paquin and Dupérré 2009). We hypothesize that two primary lineages exist in a larger “Texas eyeless” clade, but much denser taxonomic and phylogenomic sampling is needed to rigorously test this idea, particularly sampling of more eyeless species from west Texas and northeastern Mexico. We note that Cokendolpher (2004) also hypothesized common ancestry for members of the R clade, based on comparative analysis of morphology.

The capture-based DNA sequencing strategy implemented here provides a foundation for ultimately collecting nuclear phylogenomic data for all described eyeless *Cicurina* species in Texas. Collection of such data would be extremely important for testing existing species hypotheses, many of which are based on limited material and represent fundamentally weak hypotheses (see Paquin and Dupérré 2009). Also, although collection of UCE data from “standard” museum specimens was not successful in all cases (Suppl. material 5), it is clear that such samples will represent an important resource for capture-based phylogenomics research moving forward. For example, it remains possible that new DNA extraction methods (e.g., Tin et al. 2014), in combination with targeted sequence capture, will ultimately allow for the collection of useable DNA sequence data from older ethanol-preserved museum specimens.

### Species sympatry

Examples of eyed- and eyeless *Cicurina* taxa from the same Texas cave are numerous (e.g., Cokendolpher 2004), but well-supported examples of eyeless taxa sympatry in the same cave are scarce. North of Bexar County, Gertsch (1992) hypothesized sympatry of *C. reddelli* and *C. buwata*, while Cokendolpher and Reddell (2001) hypothesized sympatry of *C. caliga* and *C. hoodensis*. All of these taxa are members of a “northeast” clade within the larger ME clade (Figure 1), and thus are relatively closely related, making sympatry potentially less likely. Indeed, Cokendolpher and Reddell (2001) disclaimed the *C. reddelli* / *C. buwata* case, and the species *C. caliga* and *C. hoodensis* are genetically extremely similar (Figure 3) and are possibly conspecific (see also Paquin and Hedin 2004).

Our discovery of divergent ME and R genetic lineages, with corresponding differences in degree of troglomorphy (Figure 2), helps to now explain several cases of apparently bonafide sympatry. Cokendolpher (2004) first hypothesized sympatry between *C. platypus* (unique genitalia, ME mitochondrial clade, high TI) and *C. bullis* (R clade, low TI) in the Stone Oak KFR (Figure 8). We have hypothesized sympatry in Stahl Cave (east of Accident Sink, see Figure 8) between low TI *C. brunsi* and an unidentified member of the ME clade (possibly *C. platypus*), and have convincingly shown sympatry in the northern Culebra Anticline between *C. madla* and *C. loftini* (Figs 5 and 8). This sympatry, at karst “feature 151_15”, includes spiders collected from the same very small (~ 2 × 6 meter) solutionally enlarged fracture. The three cases above all occur in Bexar County where divergent members of the ME and R come into secondary contact, typically at the edge of species distributions. Cokendolpher (2004) hypothesized that less troglomorphic R clade taxa may be younger than taxa with elongate spermathecae; our phylogenomic topologies are consistent with this idea (e.g., Figure 2), although we have not formally conducted clock analyses.

### Immatures, new populations, likely new species

The current study was partially constrained in two ways – first, we often used immature specimens, again because about 90% of collected eyeless specimens are immature (Cokendolpher 2004, Paquin and Dupérré 2009). We felt that return on investment by inclusion was higher than complete exclusion. Also, we did not have access to all specimens borrowed from TTU (some loans were DNA only), some of which were adults. Acknowledging these limitations, our results provide important new population or species-level information in several cases, even when based on immature specimens. Put simply, our findings offer new hope in increasing the systematic information content of 100s of “unidentifiable” immature eyeless *Cicurina* that reside in regional collections (e.g., see records in Cokendolpher 2004, Paquin and Dupérré 2009).

We have shown that immatures of ME and R clade species from Bexar County differ in TI index (Figure 2), which implies that many immature museum specimens (above a certain instar) might be broadly placed into these primary lineages. This could be useful in conservation-focused identifications, or cases of sympatry where the relative abundance of sympatric individuals remains largely unstudied because of low adult spider sample sizes.

Within the ME clade, immature spiders from Bob Clark Cave (Bandera Co.) and Fern Cave (Medina Co.) represent a *potentially* undescribed sister species of *C. madla*, herein called C. cf. madla (Figure 5). This result is supported by high and congruent divergence in both nuclear (Figure 5) and mitochondrial genomes (Figure 3). We are unaware of adult spiders from these caves, but hypothesize an ME morphology similar to *C. madla*. We stress *potentially*, as these cave samples might alternatively represent new populations of an already described western species (e.g., *C. watersi*, *C. obscura*, etc. – see Paquin and Dupérré 2009, fig. 135). Collection of samples from cave habitats in the geographic region between the Government Canyon KFR and the above more westerly cave locations (Figure 5) should be prioritized.

Within the R clade, immature spiders from the “Oblate Pit Sexton” location clearly represent a second known population for the federally endangered species *C. baronia* (Figs 3, 8, Suppl. material 4), possibly indicating a previously undocumented karst connection within the Alamo Heights KFR. Robber Baron Cave is surrounded in the urban matrix of San Antonio, and as such is highly impacted (Cokendolpher 2004). The Oblate Pit Sexton location, and any karst features in the adjacent area, become extremely important from a conservation perspective. As discussed above, the species status and phylogenetic placement of immature specimens from Genesis, GreenMtn and Tuscany Heights locations requires further study (Figure 8; Suppl. material 4). Collection of adults from these locations, and sampling of phylogenomic data from more regional caves would help clarify the status of both potentially undescribed and described R clade taxa (*C. bullis*, *C. neovespera*, *C. baronia*; Figure 8; Suppl. material 4).

### The status of federally-listed *C. vespera* and *C. venii*

**Figure 11.**
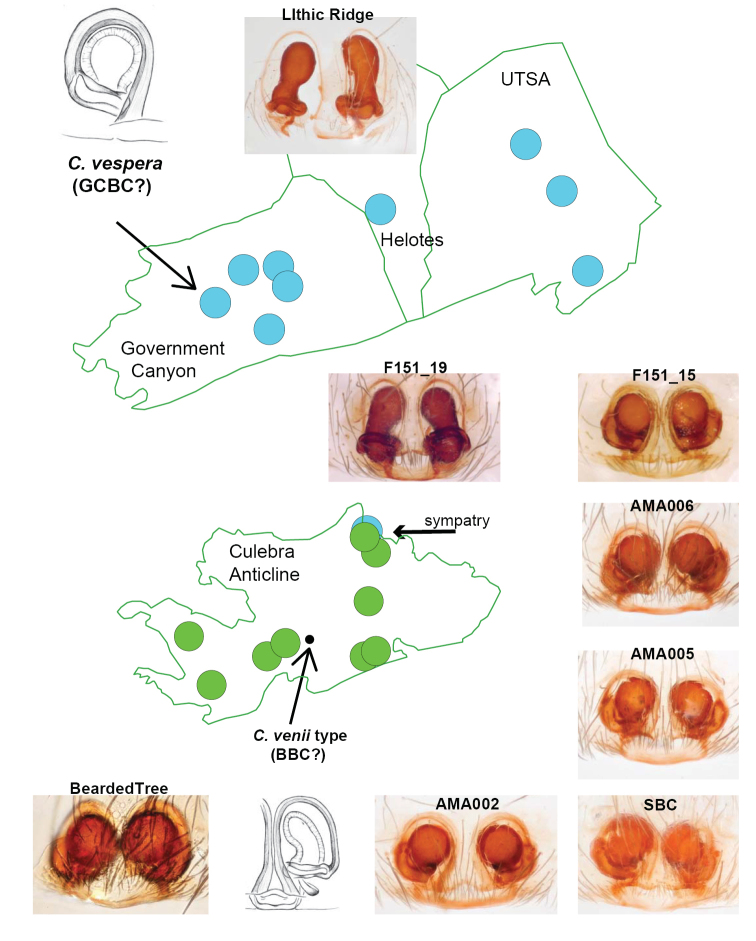
Anomalous geographic distribution of *C. venii* and *C. vespera* type specimens. All views ventral.

Both *C. vespera* and *C. venii* are single-site endemic species listed as US federally endangered (Figure 11). The female morphology and TI index of the *C. vespera* holotype (type location stated as GCBC) is like *C. loftini* (R clade), although GCBC is surrounded by *C. madla* populations (ME clade). Of seven total specimens collected from GCBC for which we have either morphological or genetic data, all are allied with *C. madla*, except for the holotype specimen of *C. vespera*. In fact, the *C. vespera* holotype remains the single known R clade specimen known from the Government Canyon, Helotes, or UTSA KFRs, all with caves inhabited by *C. madla*.

Conversely, the female morphology and TI index of the *C. venii* holotype (type location stated as Bracken Bat Cave) is like *C. madla* (Cokendolpher 2004), although Bracken Bat is surrounded by *C. loftini* populations, including other caves within 100s of meters from this location (Figs 10 and 11). Because the entrance of Bracken Bat has been sealed, additional samples from this cave for genetics research have never been available.

A key to resolving the above anomalous morphological and distributional data would have been successful collection of nuclear UCE data (or by-catch COI data) from the type specimens. We were unable to secure such data from the old holotype specimens. Given this lack of direct evidence for species status of the anomalous holotypes, we propose two alternative scenarios: 1) Gertsch (1992) either switched the labels or specimens in vials, or 2) both anomalous geographic cases represent additional examples of ME and R clade sympatry. Under either hypothesis, the species synonymies proposed below remain valid.

### Taxonomy

#### Family Hahniidae Bertkau, 1878

##### Genus *Cicurina* Menge, 1871

###### Subgenus Cicurella Chamberlin & Ivie, 1940

####### Cicurina (Cicurella) madla

Taxon classificationAnimaliaAraneaeHahniidae

Gertsch, 1992

Figs 1 , 2 , 3 , 4 , 5 , 6 , 10 , 11 , 12 , Suppl. materials 1 , 2 , 3 , 5 , 6.

Cicurina madla Gertsch, 1992: 109, figs 91–92.
Cicurina madla Cokendolpher, 2004: 42, figs 7, 40–47.
Cicurina madla Paquin & Dupérré, 2009: 28, figs 50–51, 134–135.
Cicurina venii Gertsch, 1992: 111, figs 95–96; **syn. n.**
Cicurina venii Cokendolpher, 2004: 52, figs 63–64; **syn. n.**
Cicurina venii Paquin & Dupérré, 2009: 52, figs 116–117; **syn. n.**

######## Diagnosis.

The adult morphology of a potentially undescribed sister species (C. cf. madla) remains unknown. ME clade members from the neighboring Stone Oak KFR (*C. platypus*, *C. puentecilla*) have different spermathecal morphologies (large, even-sized, rounded; Cokendolpher 2004, Paquin and Dupérré 2009). *Cicurina madla* is easily distinguished from neighboring or sympatric R clade members using genetic data, spermathecal morphology, male palpal morphology, or TI index.

######## Description.

**Figure 12.**
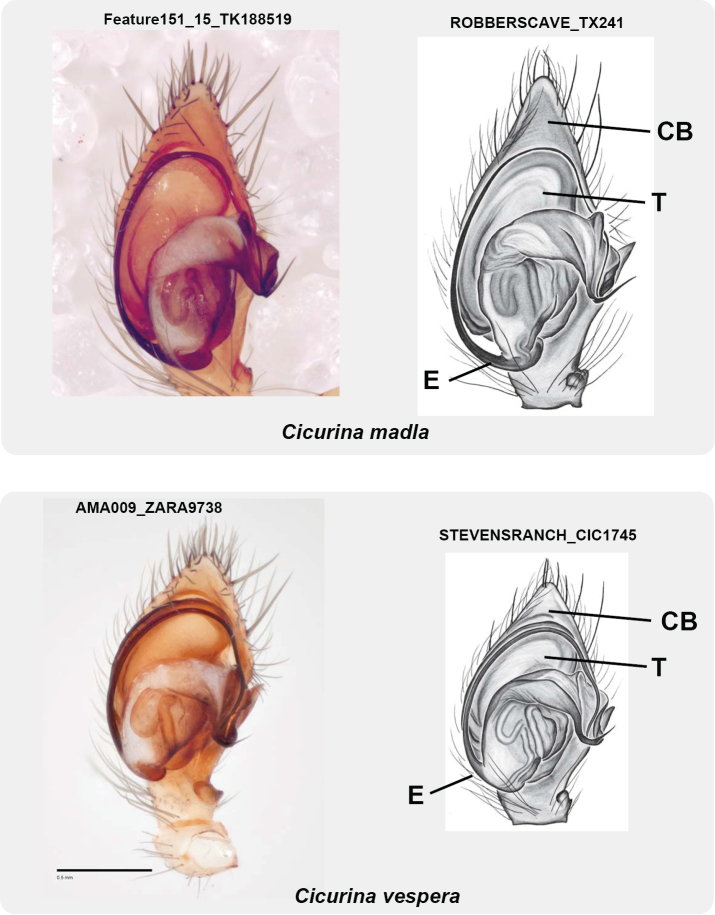
Male palpal morphology of *C. madla* and *C. vespera* (left palp, ventral view). Specimens from Stevens Ranch Trash Hole Cave (CIC_1745) and Robbers Cave (TX_241) from PP personal collection. Image of specimen TK_188519 from Joel Ledford. Abbreviations: CB = cymbium, T = tegulum, E = embolus. Scale bar: 0.5 mm.

Female spermathecal morphology as described in Paquin and Dupérré (2009). Male palpus with relatively narrow, elongate cymbium, oblong tegulum, origin of embolus at ~ 6 o’clock (Figs 6, 12).

######## Distribution.

ME clade member known from approximately 25 caves or karst features in the Government Canyon, Helotes, UTSA and northern Culebra Anticline KFRs (Figure 5), plus two populations in Stone Oak KFR and Uvalde County (Figure 6, also figure 4 of Paquin and Hedin 2004).

######## Discussion.

The high TI index, elongate spermathecae holotype *C. venii* type specimen is either 1) actually from GCBC, but was mislabeled or placed into an incorrect vial, or 2) is actually from Bracken Bat Cave, and represents a further southern (but currently unknown) extension of the *C. madla* Culebra Anticline subclade (Figure 11). If the latter, sympatry with *C. vespera* is likely in this central region of the Culebra Anticline.

####### Cicurina (Cicurella) vespera

Taxon classificationAnimaliaAraneaeHahniidae

Gertsch, 1992

Figs 1 , 2 , 3 , 7 , 8 , 9 , 10 , 11 , 12 , Suppl. materials 1 , 2 , 3 , 4 , 5 , 6.

Cicurina vespera Gertsch, 1992: 111, figs 93–94.
Cicurina vespera Cokendolpher, 2004: 53, figs 65–66.
Cicurina vespera Paquin & Dupérré, 2009: 53, figs 118–119, 134.
Cicurina loftini Cokendolpher, 2004: 41, figs 5, 10, 37–39; **syn. n.**
Cicurina loftini Paquin & Dupérré, 2009: 27, figs 46–47, 134; **syn. n.**

######## Diagnosis.

Based on well-supported nuclear phylogenomic analyses (Figs 1–2, 7), sister taxon to a clade including *C. baronia*, *C. neovespera*, and *C. bullis*. *Cicurina vespera* is morphologically very similar to the above taxa (Figure 7, Cokendolpher 2004, Paquin and Dupérré 2009), best separated by geographic allopatry (Figure 8). This species can be distinguished from neighboring or sympatric ME clade members using genetic data, spermathecal morphology, male palpal morphology, or TI index.

######## Description.

Female spermathecal morphology as described in Paquin and Dupérré (2009) and Cokendolpher (2004). Male palpus with relatively broad, truncate cymbium, compact tegulum, origin of embolus rotated slightly clockwise from 6 o’clock (Figs 9, 12).

######## Distribution.

R clade member known from 16 cave or karst features in the Culebra Anticline KFR (Figure 10); possibly also from GCBC.

######## Discussion.

The low TI index, rounded spermathecae holotype *C. vespera* type specimen is either 1) actually from Bracken Bat Cave, but was mislabeled or placed into an incorrect vial, or 2) is actually from GCBC, and represents a northern disjunct from most Culebra Anticline *C. vespera* populations, although we note that direct evidence for such an extension does not exist in the Government Canyon KFR (Figure 11). If the latter, rare sympatry must exist in GCBC, as other known specimens from this location represent *C. madla*.

## Supplementary Material

XML Treatment for Cicurina (Cicurella) madla

XML Treatment for Cicurina (Cicurella) vespera

## Supplementary materials

Supplementary material 1 Figure S1. SVD Quartets, 50 & 70% matrices This is an open access article distributed under the terms of the Creative Commons Attribution License (CC BY 4.0), which permits unrestricted use, distribution, and reproduction in any medium, provided the original author and source are credited.Marshal Hedin, Shahan Derkarabetian, Jennifer Blair, Pierre Paquin

Supplementary material 2 Figure S2. ASTRAL, 50 & 70% matrices This is an open access article distributed under the terms of the Creative Commons Attribution License (CC BY 4.0), which permits unrestricted use, distribution, and reproduction in any medium, provided the original author and source are credited.Marshal Hedin, Shahan Derkarabetian, Jennifer Blair, Pierre Paquin

Supplementary material 3 Figure S3. RAxML, 50% matrix This is an open access article distributed under the terms of the Creative Commons Attribution License (CC BY 4.0), which permits unrestricted use, distribution, and reproduction in any medium, provided the original author and source are credited.Marshal Hedin, Shahan Derkarabetian, Jennifer Blair, Pierre Paquin

Supplementary material 4 Figure S4. Relationships within *C. baronia* + *C. bullis*_*C. neovespera* clade This is an open access article distributed under the terms of the Creative Commons Attribution License (CC BY 4.0), which permits unrestricted use, distribution, and reproduction in any medium, provided the original author and source are credited.Marshal Hedin, Shahan Derkarabetian, Jennifer Blair, Pierre Paquin

Supplementary material 5 Table S1. Voucher and genetic information This is an open access article distributed under the terms of the Creative Commons Attribution License (CC BY 4.0), which permits unrestricted use, distribution, and reproduction in any medium, provided the original author and source are credited.Marshal Hedin, Shahan Derkarabetian, Jennifer Blair, Pierre Paquin

Supplementary material 6 Table S2. Troglomorphy Index data This is an open access article distributed under the terms of the Creative Commons Attribution License (CC BY 4.0), which permits unrestricted use, distribution, and reproduction in any medium, provided the original author and source are credited.Marshal Hedin, Shahan Derkarabetian, Jennifer Blair, Pierre Paquin
